# Making sense of information about HPV in cervical screening: a qualitative study

**DOI:** 10.1038/sj.bjc.6602312

**Published:** 2005-01-11

**Authors:** J Waller, K McCaffery, J Nazroo, J Wardle

**Affiliations:** 1 Department of Epidemiology and Public Health, Health Behaviour Unit, Cancer Research UK, UCL, 2-16 Torrington Place, London WC1E 6BT, UK; 2 Screening and Test Evaluation Program, School of Public Health, Edward Ford Building (A27), University of Sydney, NSW 2006, Australia; 3 Department of Epidemiology and Public Health, UCL, 1-19 Torrington Place, London WC1E 6BT, UK

**Keywords:** public understanding, human papillomavirus, cancer screening

## Abstract

Introducing human papillomavirus (HPV) testing into cervical cancer screening has the potential to change the way that women understand cervical cancer, the psychological impact of abnormal screening results and the likelihood of future participation in screening. The study used in-depth interviews to examine how women make sense of information about HPV in the context of cervical cancer screening. A total of 74 women were recruited following participation in HPV testing. Women varied widely in their beliefs about the aetiology of cervical cancer and its relationship with sexual activity, as well as in their understanding of the sexually transmitted nature of HPV. While some women who understood that HPV is sexually transmitted were able to integrate this into their existing model of cervical cancer, others were shocked by the link between cervical cancer and sex, of which they had been previously unaware. Women were generally reassured to know that HPV is common, has no symptoms, can lie dormant for many years, can clear up on its own and need not raise concerns about transmission to sexual partners. Women's understanding of HPV varied considerably, even after participation in testing. The way in which information is presented to women will be crucial in minimising the negative psychological impact of testing positive and ensuring that participation in screening remains high.

With the establishment of the role of high-risk types of human papillomavirus (HPV) in the aetiology of the majority of cervical cancers (IARC, 1995; Bosch *et al*, 2002), there is now widespread support for HPV testing in cervical cancer screening (Solomon *et al*, 2001; Wright *et al*, 2002, 2004; Cuzick *et al*, 2003). Human papillomavirus testing is recommended in the US consensus guidelines for triaging women with borderline or mildly abnormal cytology results (Wright *et al*, 2002), and endorsed by the Food and Drug Administration in primary screening for women aged 30 and over (Food and Drug Administration, 2003). In addition, encouraging research into vaccine development makes immunisation against the virus a realistic possibility in the future (Koutsky *et al*, 2002).

Such major changes to the way that cervical cancer is prevented will inevitably be accompanied by a shift in public understanding of the disease. Linking cervical cancer to a potentially stigmatising sexually transmitted infection (STI) could affect uptake of cervical screening, the impact of abnormal results (McCaffery *et al*, 2003), informed participation among women taking part in HPV screening (General Medical Council, 1998) and public acceptance of a future vaccination programme (Garland, 2003). At present, very few people in the UK know that cervical cancer is caused by HPV, and a large segment of the population seems unaware of any link between cervical cancer and sexual activity (Waller *et al*, 2004b). Studies in other countries have also found low levels of knowledge about HPV (for a review, see Waller *et al*, 2004a).

Aligning public understanding of cervical cancer with the medical model of a viral aetiology raises questions about how people will make sense of information about the virus. In this paper, we examine the responses of women participating in HPV testing to information about the virus, to assess their understanding of HPV and the impact on beliefs about cervical cancer.

## MATERIALS AND METHODS

### Participants

A total of 74 women were recruited in two UK cities, from clinical trials of HPV testing and a colposcopy clinic where HPV testing is carried out as part of routine care. Most participants had an HPV test at the same time as their routine smear, but for some, testing was offered alongside colposcopy following abnormal cytology results.

We used nonrandom, purposive sampling (Coyne, 1997) to ensure heterogeneity with respect to age, marital status, socioeconomic background and cytology result. Women from three broad ethnic groups were included: white British, South Asian (including Pakistani, Indian and East African Asian) and African Caribbean. These groups have been shown to differ in attitudes towards sexual health (Elam *et al*, 1999) and uptake of cervical screening (Health Education Authority, 1994). Women who tested positive or negative for high-risk HPV types were included.

### Procedure

Within each centre, all women had been given standardised information about HPV, although there was some variation in the detail between centres. They were all given a leaflet informing them about the sexually transmitted nature of the virus, its high prevalence, its tendency to lie dormant and its link with cervical abnormalities as well as warts. The wording describing sexual transmission was often open to a degree of interpretation; for example one leaflet said ‘Although there may be occasional exceptions, it is thought that Human Papillomavirus is sexually transmitted’. Some women also called help-lines or consulted health professionals for additional information.

In-depth interviews were carried out with women, usually in their own homes. These were structured around a topic guide covering general background, screening history, beliefs about cervical cancer, understanding of HPV, experience of participation in HPV testing, and understanding and impact of results. Interviews were carried out by the first and second authors and by trained, freelance interviewers, using a mixture of ethnic matching and mismatching, and were translated where necessary. They were transcribed verbatim.

Analysis used the Framework method to identify emerging themes and organise the data (Ritchie *et al*, 2003). This is a matrix-based approach, with themes making up the columns and cases making up the rows. The thematic framework is developed through familiarisation with a subset of transcripts. All data are summarised within the framework and this organisation facilitates examination of both themes and cases, allowing relationships and explanations for patterns within the data to be explored. Detailed analysis was carried out by two researchers (JW and KM) and interpretations were checked and discussed with a third team member (JN).

The study was approved by the North West Medical Research Ethics Committee.

## RESULTS

The demographic characteristics of the sample are shown in Table 1.

### Causes of HPV and cervical cancer

A typology was developed to capture the range of women's causal conceptions of cervical cancer, HPV and the relationship between the two, with a specific focus on sexual activity as a risk factor. This is shown in Box 1 with each type detailed below.

#### 1. Causal conceptualisations absent for cervical cancer and HPV

Women in this group did not talk about cause in relation to cervical cancer or to HPV. They tended to know little about cervical cancer and had very little awareness of HPV. Testing negative for HPV, or having the test as part of the management of an abnormal smear, seemed to be associated with this lack of conceptualisation, although it was also seen among HPV-positive women with serious abnormalities. One example was a South Asian woman who tested negative for HPV. When asked about what caused cervical cancer, she said:

Oh God. I don't know (laughs). I don't think I've ever thought of it in that sense. SA/N1* HPV− smear normal

When asked why she thought some people might get HPV, she said ‘I don't know’. Having always had clear smear results in the past, she seemed never to have thought about what might cause an abnormality, and had not read about HPV as her result was negative.

#### 2. Cervical cancer unrelated to sex

Women in this group had some awareness that HPV is linked to sexual activity. Although most knew that it could be an STI, other causal explanations were given, and there was widespread belief that HPV was somehow different from other STIs. This group was also characterised by a lack of awareness of the link between cervical cancer and sexual activity. Other models of cancer were put forward, with causes including bad luck, chance, family history and smoking. The link between HPV and cervical abnormalities or cancer was generally poorly understood, so women in this group were able to retain their model of cancer as something that could not be linked to sex.

A clear example is a white woman who did not link attendance at cervical screening with being sexually active, and when asked directly whether she believed that sex could affect cervical cancer risk, she said no; she thought it ‘just appeared’. She was uncertain about the sexually transmitted nature of HPV, and did not think that the link between HPV and cervical cancer had been established. This quote illustrates the contrast between the information about HPV and her existing model of cervical cancer:

I have never thought that you catch cervical cancer through having too much sex. I don't know, that seems laughable because cancer is such a big word and sex is quite pleasurable so you wouldn't expect to get something like that from it. W/P13 HPV+ smear normal

Although she was aware of the idea that HPV might be sexually transmitted, by doubting the connection she was able not only to manage her own response to her positive result (‘if it is sexually transmitted I think I'd feel quite sick’), but also to maintain her current model of cancer as something that does not have easily identifiable causes.

Beliefs in this group were sometimes very complex. Another woman (W/P2, HPV+ borderline smear) knew that attending for cervical screening was associated with the onset of sexual activity, but had a very clear model of cancer: ‘I think everyone's got cancer in them and it takes … something to set it off, to wake it up’. Although she had been told that HPV is sexually transmitted, she did not conceptualise it as being similar to other STIs. She understood that HPV was linked to cancer, which she did not see as being something you catch and this led her to believe that you could get HPV in other ways: ‘I think it wasn't like a specific thing to, to sex. … I don’t know, I suppose, with it being cancer … I wouldn't … associate it with [being caught from] anyone else’.

In some cases, women were clearly aware that HPV is an STI, but they did not conceptualise it as causally related to cervical cancer, so, again, were able to maintain their model of cervical cancer separate from sexual transmission.

#### 3. Cervical cancer possibly related to sex

All the women in this group mentioned the link between cervical cancer and some aspect of sexual activity, be it having many partners, having sex when young or nuns having low rates of the disease. However, they did not see it as inevitably linked to sex, and held other concurrent causal beliefs. Human papillomavirus was also linked with sex to some extent, but the causal relationship between HPV and cervical cancer was not recognised, or had not been thought through sufficiently to lead the women to reassess their beliefs about cervical cancer. Many of the women in this group, and in the group below, believed that the research in which they were taking part was designed to investigate a possible link between cervical cancer and HPV.

A nurse in this group cited having many sexual partners as an established risk factor for cervical cancer, but seemed unable to reconcile her model of cancer with her beliefs about sexually transmitted diseases. She knew that HPV is an STI, but regarded HPV as very different from cervical cancer:

Well, cervical cancer … that's not just caused by a sexually transmitted disease. Anyone can, except that, well, no, you're not going to, you're not likely to get cervical cancer if you've never been sexually active, so it is, it is only in, in sexually active people but [Sighs] Well, I don't know, it is, it's just different. Because that's, I mean cervical cancer … is a killer. The HPV is a, a nuisance. W/P6 HPV+ CIN1

In this quote, she seems to be struggling to bring together her medical knowledge about the risk factors for cervical cancer with her lay belief that cancer and STIs are unrelated.

Other women talked about sexual activity not being the ‘sole cause’ of cervical cancer, or about knowing people who had had the disease, and it not being related to sex in their case. This woman talked about a friend who was diagnosed with cervical cancer:

The doctors used to say ‘Oh if you have more than a certain amount of sexual partners’ blah blah blah but … I know that she was a virgin when she went with [boyfriend's name], so I know she wasn't one of those promiscuous women so then I thought that's a load of crap. AC/P7 HPV+ CIN2-3

These women did not conceptualise the relationship between HPV and cervical cancer clearly enough to necessitate a reassessment of their beliefs.

#### 4. Both cervical cancer and HPV linked to sexual activity

This group was similar to the previous group, except that they held firmer beliefs about the causal relationship between sex and cervical cancer, often providing explanations for the mechanism through which this might operate: ‘something to do with the sperm that can damage the cervix’ (W/P12 HPV+ smear normal); ‘some infection that you could pick up at that young age which may start that off’ (W/P33 HPV+ CIN2-3); ‘young boys … can be quite rough [during sex]’ (W/P27 HPV+ CIN2-3). They were aware of the possibility that HPV could be sexually transmitted, although not all believed it. This, together with uncertainties about the relationship between HPV and cervical cancer, prevented them from fully integrating their models of HPV and cervical cancer.

A Caribbean participant held a variety of beliefs about how the relationship between cervical cancer and sex might operate: through ‘unprotected sex’, ‘infections’ and ‘lots of bashing’. She was aware that HPV could be transmitted by sexual partners, but had not fully understood the causal link between HPV and cervical cancer. She said she knew there was a connection between the two, but she did not know what it was. Despite knowing that both cervical cancer and HPV are related to sex, she did not apply this to her own abnormal smear and HPV-positive result:

Did I think the whole thing was sex? (Pause) Not necessarily. I don't know. I just thought cancer. (Pause) Yeah because you can get HPV it doesn't necessarily affect guys as it does women in the same way and it can actually be transmitted through … sexual partners and stuff. Hence the reason they ask about sexual partners [when you go for a smear] and blah-di-blah and protection and all that kind of stuff. … I didn't feel ‘Oh my God I feel dirty and unclean’ ‘cause I didn't associate the HPV as much to the sexual thing. I was thinking more of the cancer thing. AC/P4 HPV+ CIN2-3

Throughout the interview, she expressed a series of slightly contradictory beliefs, clearly trying to make sense of the information about HPV in the context of her pre-existing beliefs about cervical cancer.

#### 5. Little or nothing known about HPV

Women in this group described a variety of beliefs about the relationship between cervical cancer and sexual activity – some held sophisticated causal models while others doubted the relationship was true. The group was characterised by a lack of understanding of HPV, and particularly by a lack of awareness that it is sexually transmitted. One reason for the lack of awareness of HPV was that many of these women had tested negative for the virus, or had not received their results at the time of the interview. This meant that they had not received detailed information about HPV, or had not felt the need to read it as their result was normal. Other women received the HPV result at the same time as a smear result and interpreted it as being another test for the same thing.

An example was a woman who tested positive for HPV but had a normal smear result (W/P22 HPV+ smear normal). She had heard of a link between cervical cancer and number of sexual partners, but did not think that it could be true, believing that cancer ‘can hit anyone’. She believed that HPV was ‘something connected with the smear’ and so thought that ‘it's like cervical cancer; it can hit you at a certain point in life’. In the absence of any other knowledge about HPV, she seemed to extrapolate from her beliefs about cervical cancer to construct causal attributions for the virus.

#### 6. Recent awareness of the link between cervical cancer and sexual activity

This group was characterised by a lack of previous understanding of the link between sexual activity and cervical cancer. Information about HPV had therefore forced a change in women's model of cancer, which was often associated with feelings of shock. Women in this group were different from those in previous groups, in that they had read and understood the information about HPV and had often sought additional information, frequently leading to a sophisticated understanding of the virus and its link with cervical cancer. A white woman described her reaction when she realised that cervical cancer was caused by a sexually transmitted virus:

When I got the HPV I was horrified to discover that sex could lead to cancer. Yeah. So that was new information to me. I remember the shock of discovering that and thinking everybody should know about this. It's really shocking. W/P17 HPV+ CIN2-3

A South Asian woman expressed similar views and wished that she had known sooner about the link in order to make changes to her behaviour once she had HPV:

I actually did a lot of research and it was only then that I realised that it is actually sexually related because I was under the impression that it was just my cells going crazy in me, having cancer all of a sudden. … The information about where it actually originates and how you can deal with it can help a lot. … I would have … insisted on, say, condoms … so it would have made a world of difference. SA/P7 HPV+ CIN2-3

Many of these women expressed a sense of surprise and disbelief that they had never heard about the link between cervical cancer and sexual activity before. They were frequently well-educated women who took an interest in health issues, and were astonished that they had never heard of it. For some, there was a sense of anger that doctors must have been aware of a link between cervical cancer and sex, but that this information was not in the public domain, in what one woman described as a ‘conspiracy of silence’.

#### 7. Longstanding awareness of the link between cervical cancer and sexual activity

This group was made up of women who had previous awareness of the link between sexual activity and cervical cancer, and who were able to integrate the new information about HPV into their existing causal framework: ‘I always understood that one of the exacerbators of cervical cancer or the promoters, facilitators of cervical cancer was not only your own body malfunctioning at random, was also the nun syndrome’ (W/P4 HPV+ smear normal). The extent to which cervical cancer was therefore seen as a sexually transmitted disease, and to which this was made explicit in the interview, varied.

A white woman described her longstanding knowledge that the incidence of cervical cancer is lower in ‘Jewish ladies’ and nuns. This made her think that ‘it's obviously down to some kind of sexual preference and possibly foreskin’. However, the information about HPV made this much more explicit, and caused a shift in her model of cervical cancer towards a sexually transmitted aetiology:

Before I heard about this [HPV] I would have just classed [cervical cancer] as the same form of cancer. Since I've heard about this and talked to people I would say it's totally dissimilar to any other kind of cancer. … It almost feels like it's more of, like a sexual transmitted disease. It's that, it's picking up something off a sexual partner that's causing the cancer, where there's no other kind of cancer that you can catch, basically, from somebody else. W/P30 HPV+ smear normal

This account illustrates the way in which cervical cancer is now conceptualised as being more similar to a sexually transmitted disease than to other cancers.

### Other HPV knowledge

The preceding typology has illustrated the way in which women's models of cervical cancer might be affected by information about HPV. If screening is introduced and information about HPV becomes more widely available, it is likely that most people will shift into typologies 6 and 7, understanding the relationship between an STI and cervical cancer, either as a new concept or within an existing causal framework. In this sample, understanding the sexually transmitted nature of HPV was associated with psychosocial responses typical of other STIs, like stigma, blame or problems with disclosure. These are described in detail elsewhere (McCaffery *et al*, 2004b). It appeared that certain other aspects of HPV knowledge were key to minimising the potential negative impact of a positive result, and these are summarised briefly below.

#### High prevalence

The information leaflets that women received told them that HPV is very common. Reading this after testing positive for HPV, or having this information reinforced by clinicians, seemed to reduce the perceived stigma as well as the perceived severity of HPV. This woman found the information on the internet:

The research that I got back was like seventy five per cent of women have it and don't realise it. I suddenly thought why, why is it such a stigma then? W/P31 HPV+ CIN2-3

#### Lack of symptoms

There was some confusion about whether high-risk HPV has symptoms or not, with some women expecting that they might develop genital warts. This added to the distress caused by a positive result. Knowing that there are no symptoms allowed women to ‘go on with everyday life and not think about it’ (W/P13 HPV+ smear normal).

#### Spontaneous clearance

Women were reassured by the fact that HPV can ‘clear up’ on its own and that treatment is not normally necessary. This allowed comparison with other trivial infections like colds: ‘in the majority of cases the system will clear, as in the common cold’ (SA/P3 HPV+ smear normal).

#### Dormancy

As described elsewhere (McCaffery *et al*, 2004b), many women felt ‘shocked’ or ‘horrified’ to be diagnosed with an STI, especially those who were either in a long-term monogamous relationship or who were not currently sexually active. Knowing that HPV can lie dormant for a long time without causing problems was extremely reassuring and allowed women to attribute the infection to a previous partner, or to assume that their partner had contracted it during a previous relationship. This minimised the extent to which women's current relationships were threatened by the infection, and relieved confusion about how they could have contracted HPV.

I was horrified. … I'd only ever had a relationship with my husband and I just couldn't see how I would have got a sexually transmitted disease. … I mean it could be that … my husband had had it and passed it on to me and he, he wouldn't have known, because it doesn't have, cause men a problem. It just goes away … I mean, now I understand that, you know, it can just lie dormant in women and not give them a problem, so that obviously was quite reassuring W/P6 HPV+ CIN1

#### Future transmission of the virus

The previous quote also illustrates the reassurance of knowing that HPV does not cause a problem for men and that transmission to a male partner need not be a cause for concern. Many women remained confused about this and had concerns about transmission of the virus in current or future relationships. They often wondered whether they should disclose their HPV-positive status to future sexual partners.

What kind of precautions do I need to take in having this? And obviously I can give it to other people, and is that bad if I give it to other people? W/P16 HPV+ CIN2-3

There was also uncertainty about whether condoms are protective. For some, the fact that HPV cannot be fully prevented by condom-use reduced the stigma of the infection, but among others there was a sense of helplessness that the infection could not be prevented. Most distressing seemed to be the lack of clear information and advice available.

But nobody's actually sat down and said ‘Look, you now have to be careful. You now have HPV’ and I think if somebody said ‘Look you really do have to either use condoms or inform future partners’ I would do. If somebody actually said ‘You need to start doing that’. But still nobody's really told me how serious or little of importance this virus is. W/P30 HPV+ smear normal

## DISCUSSION

This study indicates that the dissemination of information about HPV, which will inevitably accompany the introduction of HPV testing and the policy debate on vaccination programmes, has the potential to change radically the way that women conceptualise cervical cancer. Many women do not know that cervical cancer is linked to sex and may be shocked to discover that it is caused by an STI. The kind of information that is provided about HPV is likely to affect the impact of testing positive for the virus, although we have identified specific information that appears to be reassuring. Perhaps most important, and consistent with the findings of Maissi *et al* (2004), is the need for clear and consistent information about HPV to minimise the anxiety associated with uncertainty and confusion. This need is illustrated very clearly by the following quote:

This conflict of information was diabolical. It wasn't that I was reading it out of Cosmopolitan. It was the fact that I was ringing STD clinics in hospitals, different hospitals and asking their opinion and they were all giving different information. … I think that's quite a surprise ‘cause you think that you trust people in that position, they're dealing with it every day. But I rang, I think, four places and they all gave different information which suggests that maybe the information isn't there. … They don't know. So once again it's difficult to make choices if you haven't got the information. W/P17 HPV+ CIN2-3

This is the first study to use qualitative methods to gain an in-depth understanding of the perceptions of women taking part in HPV testing in the context of cervical cancer screening, rather than questioning women hypothetically (McCaffery *et al*, 2003; Anhang *et al*, 2004) or using quantitative methods to measure anxiety (Maissi *et al*, 2004; McCaffery *et al*, 2004a). It is considerably strengthened by the inclusion of women from contrasting ethnic and socioeconomic backgrounds. Although we achieved heterogeneity among our participants and representation across all the selected demographic categories, a relatively high proportion (44%, *n*=33) of our sample had University education. Further research may be needed among women with lower levels of education. Indeed, further research to quantify the typologies among a representative sample of British women undergoing HPV testing and to indicate the dominance of certain typologies over others and to examine changes over time would be useful. The qualitative method and purposive sampling used in this study make any quantification of the data inappropriate and beyond the scope of the study.

In total, the findings point to the need for the development of consistent and clear information about HPV, and identify a role for general practitioners and other clinicians in providing women with this information. One US study has begun to develop responses to frequently asked questions about HPV (Gilbert *et al*, 2003), but more work in this area is clearly needed. If we fail to address women's information needs, we run the risk of reducing participation in the currently very successful screening programme and the psychological harm of participation in screening may be severe.

## Figures and Tables

**Table 1:** Demographic characteristics of the sample (*n*=74)

**Demographic characteristics**	***n***
Age	
20–29	22
30–39	27
40–49	16
50–64	9

Relationship status	
Single	20
In a relationship (not cohabiting)	10
Cohabiting	11
Married	24
Divorced/separated/widowed	9

Education	
Left school before 16/no qualifications	10
Left school at 16 (GCSEs, CSEs, O'levels)	13
Further education (diploma, Btech, etc.)	12
Higher education (degree)	33
Missing data	6

Ethnic group	
White	41
South Asian	17
African Caribbean	16

Cytology result	
Normal	34
Abnormal	40

HPV result	
Positive	57
Negative	17

**Box 1:** Typology of beliefs about the relationship between cervical cancer, HPV and sex

1.	Causal conceptualisations were absent for both cervical cancer and HPV, even if a link between the two was recognised.
2.	Cervical cancer was unrelated to sex and HPV was understood to be linked with sex, but the relationship between HPV and cervical cancer was not well understood, so the existing model of cervical cancer was maintained.
3.	Cervical cancer was seen as possibly related to sex, but other causal models were also held. Human papillomavirus was understood to be linked to sex, but implications for the sexually transmitted nature of cervical cancer were not drawn.
4.	Both cervical cancer and HPV were linked to sexual activity, but implications for the sexually transmitted nature of cervical cancer were not explicitly recognised.
5.	Little or nothing was known about HPV, although the link between cervical cancer and sexual activity was understood.
6.	Recent awareness of the link between cervical cancer and sexual activity since receiving information about HPV led to a reconceptualisation of cervical cancer as linked to sex.
7.	Longstanding awareness of the link between cervical cancer and sexual activity meant that information about HPV was compatible with pre-existing beliefs and could be integrated into the existing causal framework.

## Footnotes

* Participant ID numbers use W, SA and AC to denote White, South Asian and African Caribbean women, respectively, and P or N to denote HPV positive and HPV negative.
